# Cervical vestibular evoked myogenic potentials in healthy children: Normative values for bone and air conduction

**DOI:** 10.3389/fneur.2023.1157975

**Published:** 2023-04-18

**Authors:** Sylvette R. Wiener-Vacher, Marta Campi, Priscilla Boizeau, Hung Thai-Van

**Affiliations:** ^1^Institut de l’Audition, Institut Pasteur, CERIAH, Paris, France; ^2^Service ORL, Centre d’Exploration Fonctionnelle de l’Equilibre chez l’Enfant (EFEE), Hôpital Universitaire Robert-Debré AP-HP, Paris, France; ^3^Unité d’Epidémiologie Clinique, INSERM CIC1426, Hôpital Universitaire Robert-Debré AP-HP, Paris, France; ^4^Hospices Civils de Lyon, Hôpital Edouard Herriot & Hôpital Femme Mère Enfant, Service d’Audiologie & Explorations Oto-Neurologiques, University of Lyon, Lyon, France

**Keywords:** otolith, vestibular evaluation, pediatric, age, vestibulospinal pathway, data modeling

## Abstract

**Objectives:**

To characterize cervical vestibular evoked myogenic potentials (c-VEMPs) in bone conduction (BC) and air conduction (AC) in healthy children, to compare the responses to adults and to provide normative values according to age and sex.

**Design:**

Observational study in a large cohort of healthy children (*n* = 118) and adults (*n* = 41). The c-VEMPs were normalized with the individual EMG traces, the amplitude ratios were modeled with the Royston-Wright method.

**Results:**

In children, the amplitude ratios of AC and BC c-VEMP were correlated (*r* = 0.6, *p* < 0.001) and their medians were not significantly different (*p* = 0.05). The amplitude ratio was higher in men than in women for AC (*p* = 0.04) and BC (*p* = 0.03). Children had significantly higher amplitude ratios than adults for AC (*p* = 0.01) and BC (*p* < 0.001). Normative values for children are shown. Amplitude ratio is age-dependent for AC more than for BC. Confidence limits of interaural amplitude ratio asymmetries were less than 32%. Thresholds were not different between AC and BC (88 ± 5 and 86 ± 6 dB nHL, *p* = 0.99). Mean latencies for AC and BC were for P-wave 13.0 and 13.2 msec and for N-wave 19.3 and 19.4 msec.

**Conclusion:**

The present study provides age- and sex-specific normative data for c-VEMP for children (6 months to 15 years of age) for AC and BC stimulation. Up to the age of 15 years, c-VEMP responses can be obtained equally well with both stimulation modes. Thus, BC represents a valid alternative for vestibular otolith testing, especially in case of air conduction disorders.

## Introduction

Vestibular evoked myogenic potentials (VEMPs) allow an objective evaluation of vestibulospinal pathway function. Although neck muscle responses to acoustic stimuli were reported in humans as early as 1963 by Kiang (1), their vestibular origin was not claimed. The first detailed study of VEMPs in humans was published in 1994 by Halmagyi et al. (2), and has been followed by numerous publications (3–7). This technique is based on the stimulation of otolith receptors by high amplitude, low frequency acoustic stimuli delivered either by air conduction (AC) or bone conduction (BC), as demonstrated by studies conducted in experimental animals (8, 9). These stimuli activate the vestibulospinal pathways, triggering robust evoked myogenic potentials at the level of the neck muscles, described as cervical VEMPs (c-VEMPs). Research conducted in animals found that AC stimulates preferentially saccular receptors, while BC stimulates both saccular and utricular receptors (9, 10). While this technique, particularly with AC stimulation, has been widely used in adults (6, 11), a few studies later reported its adaptation to children (12–16). It has even been reported that AC c-VEMPs can be recorded in term (17) and pre-term newborns (18), but not with a 100% success rate. BC c-VEMPs have been successfully used as a vestibular screening test in a large group of hearing-impaired infants tested at the age of 6 months (19) and the feasibility of BC c-VEMPs in a newborn hearing screening protocol has also been demonstrated (20). A decrease in AC c-VEMP amplitude response with age has been reported in healthy adult subjects (3, 4, 21), as well as in a wider population of 85 healthy participants (10 patients aged 10–19 years) for both AC and BC c-VEMP amplitude responses (16). In another study conducted in a population including children older than 5 years (*n* = 21) and adults (*n* = 9) c-VEMP amplitude was observed to decrease with age for BC but not AC (22). One study of 30 healthy children aged 3–11 years reported shorter AC c-VEMP latencies only in participants aged from 3 through 5 years (23).

However, it is not known whether AC and BC produce c-VEMP with equivalent characteristics for the whole population with respect to age and sex. The objectives of this study were to characterize BC and AC c-VEMPs in healthy children, to compare them to adults, and to provide their normative values as a function of age and sex.

## Materials and methods

### Population

**Figure 1:**
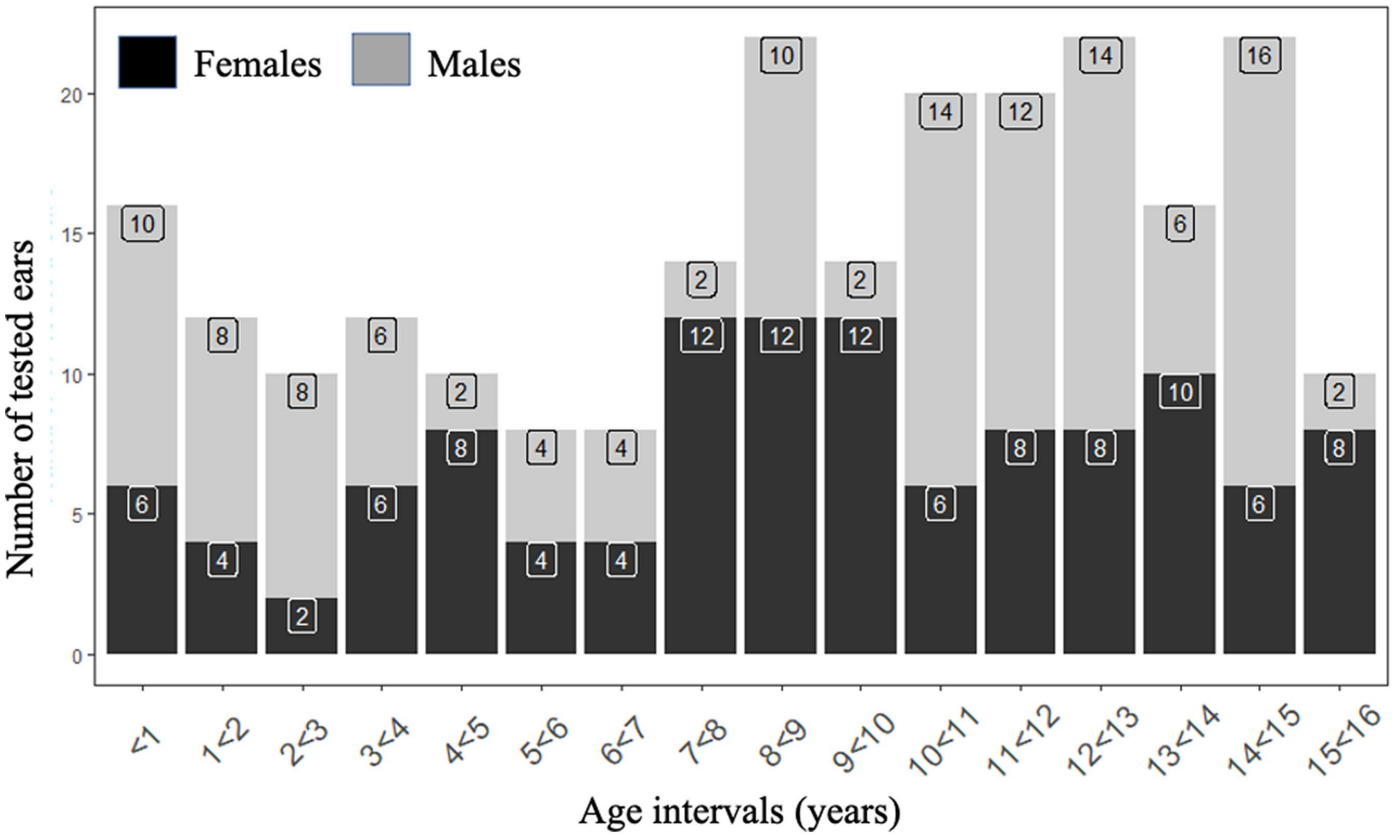
Age and gender distribution of the population of healthy children and adults.

The child group included 118 subjects aged 2 months to 15 years and 11 months, including 8 infants (<1 year old): one 2 months old and the others older than 6 months (Figure 1). There were 60 boys and 58 girls (Figure 1). All children had a pediatric follow-up since birth that did not reveal any developmental abnormalities. Their otorhinolaryngological (ENT), neurological and vestibular clinical examinations, performed by a skilled neuro-otologist, was normal. At the time of inclusion, they all passed an audiometric test adapted to their age, had transient otoacoustic emissions present on both ears, and normal middle ear function as evidenced by type A tympanometry (peak between ±100 daPa). The adult group included 41 subjects aged 16–61 years (mean ± SD: 35.5 ± 15 years) with 11 males and 30 females. All adult participants had normal ENT, neurological and vestibular clinical examinations, as well as normal pure tone audiometric thresholds at 0.5, 1, 2, 4, 8 kHz, and type A tympanometry (peak between ±100 daPa). Subjects were tested for AC and BC c-VEMP on both ears (children: 236 ears, adults: 82 ears). Consent forms were obtained from all parents and from adult subject in adherence with the principles of the Declaration of Helsinki and the study was approved by the relevant institutional human experimentation committee (Comité de Protection des Personnes, Ile de France; # 96048).

### Recording protocol

#### Stimulation parameters

**Figure 2:**
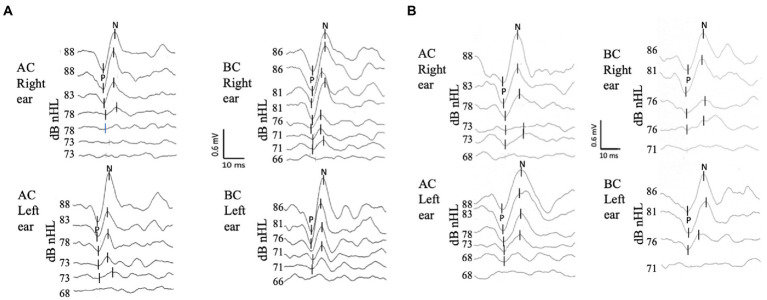
C-VEMP responses to air conduction (AC) and bone conduction (BC) stimulation for two subjects. Each trace is an average of 25 responses. P is the first peak, and N is the second peak of the biphasic c-VEMP responses. **(A)** 6-year-old child. The top traces for each side and mode of stimulation correspond to the reference level of our protocol 88 dB nHL (i.e., 100 dB HL) for AC and 86 dB nHL (i.e., 100 dB HL) for BC. Threshold (defined as the lowest level of stimulation where a replicable waveform was observed) were in this subject for AC 78 dB nHL on the right and 73 dB nHL on the left, and for BC 71 dB nHL on the right and 71 dB nHL on the left. **(B)** c-VEMP recordings for a 18 year old adult. The thresholds are in this subject: for AC 73 dB nHL s, and for BC 76 dB nHL on both sides.

The c-VEMP recordings were performed using a brainstem evoked auditory response device (Centor plus^®^, Racia Alvar, Bordeaux, France), equipped with an additional amplifier allowing high-intensity level tone-bursts to be delivered (up to 110 dB HL, i.e., 96 dB nHL for BC and 98 dB nHL for AC; see Table 1 for equivalences). Short tone bursts (4 msec duration with one cycle for rise, plateau and fall time, 750 Hz frequency, 4 pps delivery rate) were delivered for AC using headphones (TDH39, Huntington, NY, United States) and for BC, a B71 vibrator (RadioEar, New Eagle, PA, United States) applied to the mastoid ipsilateral to the tested ear. Stimulus calibration followed standard procedures. Stimuli were calibrated in dB peSPL (peak-to-peak equivalent SPL) according to IEC 60645–3:2020 standard for AC, and in dB FL for BC. In the AC condition, TDH39 headphones were calibrated in an artificial ear according to IEC 60318–1:2009 standard. For BC, the B71 bone vibrator was calibrated in an artificial mastoid according to IEC 60318–6:2007 standard. The RETSPL values (reference equivalent threshold for dB nHL scale) for tone-bursts were derived from ISO 389-6:2007 standard. The recording system allowed well-identified biphasic c-VEMP responses to be elicited in an average of 25 repetitions for each level of stimulation (Figures 2A,B). Stimulation levels started at 100 dB HL, i.e., 86 dB nHL for BC and 88 dB nHL for AC, and then decreased by 5 dB steps in order to identify the threshold that is the lowest stimulation level allowing P and N waveform to be reproducible on two successive trials. In case of no response at 86 dB nHL for BC and 88 dB nHL for AC, the stimulation level was increased by 5 dB steps up to the maximum output level of the recording system to identify the thresholds. The test conditions were standardized (AC first). Test order between ears was randomized. The standardized protocol used here was adapted for children since it required a small number of stimulations for each stimulus amplitude (*n* = 25), allowing a short test duration (<10 min), and thus improved feasibility. No data were excluded due to poor reliability, and all participants were able to complete the entire test protocol.

**Table 1:** Stimulus level conversion table.

Stimulus level for BC 750 Hz tone burst (B71)			Stimulus level for AC 750 Hz tone burst (TDH39)		
dB HL	dB nHL	dB FL	dB HL	dB nHL	dB SPL
110	96	159	110	98	119
105	91	154	105	93	114
100	86	149	100	88	109
95	81	144	95	83	104
90	76	139	90	78	99
85	71	134	85	73	94
80	66	129	80	68	89
75	61	124	75	63	84
70	56	119	70	58	79

#### Electromyographic and c-VEMP recordings and characterization

**Figure 3:**
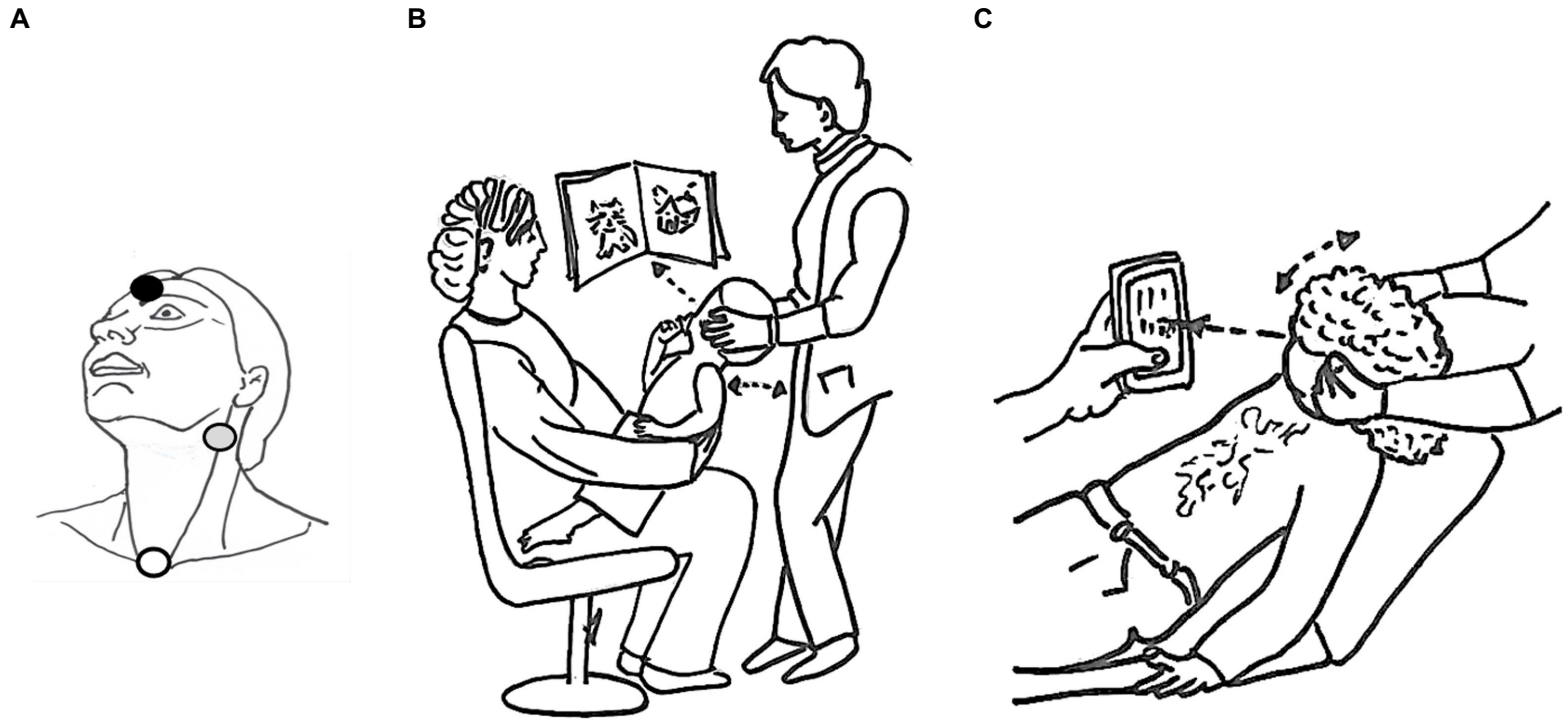
Recording protocol. The position of the surface electrodes is shown in **(A)**: active electrode in gray, reference in white and ground in black. The best c-VEMP test position is shown for children under 6 years of age **(B)** 6 years and older **(C)**.

The c-VEMP recordings were carried out using surface electrodes. The active electrode for C-VEMP was positioned at the mandibular angle on the anterior edge of the sternocleidomastoid muscle, the reference electrode on the suprasternal notch, and the ground electrode on the middle of the forehead at the nasion (Figure 3A). The EMG was averaged for each tone-burst and individual trace, within a 150 msec window starting at the onset of the tone burst. The c-VEMP peak-to-peak response was analyzed on a 50 msec window starting with the onset of the stimulus. For each recording session, all traces obtained with the same EMG level were selected offline for averaging. This allowed comparisons of c-VEMP amplitude, latency, and threshold within the same session, as well as between sessions carried out at different times (16, 24). To compensate for the great inter- and intra-individual variability of neck muscle contractions, the c-VEMP peak-to-peak amplitude was expressed relative to the EMG level as an amplitude ratio (PN/EMG) and measured at the reference stimulation level (25). For each recording session, the c-VEMP responses were analyzed for amplitude (P peak to N peak amplitude, P-N), latency (P latency for the first peak and N latency for the second peak), and threshold, and these were used to characterize age and sex differences. The inter-aural relative asymmetries were calculated from the c-VEMP the amplitude ratio ([Right ear -left ear]/[Right ear +Left ear]*100).

#### Test set-up

The best position to obtain sustained neck muscle contraction was chosen according to age. The youngest children were seated on a parent’s lap, face-to-face, while the parent was instructed to support the lowest part of the child’s back. The examiner slightly tilted the child backward, turning his/her head away from the stimulation side. This position led the child to straighten up and look at or reach for a toy presented on that side (Figure 3B). Adults and children over 6 years of age partially reclined on an examination table and were asked to lift and turn their head away from the stimulated ear (Figure 3C). Visual feedback of the EMG recording was simultaneously provided to the patient and the examiner in the form of a car speedometer displayed on a monitor, facilitating verification of patient neck muscle contraction. The EMG target range was 100–500 μV. For each stimulation level, acquisition was stopped as soon as reproducible c-VEMP responses were obtained, which usually occurred after 25 repetitions in children and 50 repetitions in adults. The number of acquisitions was systematically increased up to 50 either for threshold determination, or when no reproducible responses were obtained at the maximum stimulation level. The recordings at threshold were reproduced at least twice for validation. To compare between subjects, we choose as a reference value, responses to stimulations at 100 dB HL (i.e., 86 dB nHL for BC and 88 dB nHL for AC). There, the responses were clear for almost all patients c-VEMP parameter. This stimulation was reproduced twice for validation.

### Data analyses

The c-VEMP characteristics were compared between children and adults with a Wilcoxon Mann–Whitney test for continuous data (amplitude ratio and latency), and a Cochran–Mantel–Haenszel test for ordinal data (c-VEMP threshold). For paired data (i.e., AC vs. BC for amplitude ratio, threshold and latency), the differences between the AC and BC measures followed a Gaussian distribution, allowing the use of paired *t-*tests.

To compare left- vs. right-ear responses, a paired *t-*test was performed on amplitude ratio to account for potential intra-individual variability. Agreement between left- and right-ear data was also checked with Intra-class correlation (ICC).

Comparison between independent age subgroups for the whole population required the use of non-parametric tests since the data distributions for each subgroup were not Gaussian. Estimation of age-specific reference intervals for c-VEMP amplitude ratio relied on Wright and Royston method (26, 27). This method has been recommended by the World Health Organization for the construction of growth curves (28). Using low-order fractional polynomials for each of the parameters of a modulus-exponential-normal distribution, it allowed here for calculation of mean, SD and fitting centiles according to age and sex. For this, a Box Cox transformation was first applied on the amplitude ratio values, B. Second, the means, μ, were modeled using polynomials in age, and standard deviations (SD) were then estimated. Successive steps allowed assessment of the goodness-of-fit with the model. The Z-score was calculated [*Z* = (B − μ)/σ] and must follow a normal distribution [for more detail, (27)]. The qualitative variables were described as numbers (percentages) and quantitative variables as mean ± SD or median [interquartile range, IQR] depending on whether distributions were Gaussian or not.

Descriptive statistics and statistical analyses were performed using SAS version 9.4 for Windows (SAS Institute Inc., Cary, NC, United States) and R-4.2.2 for Windows. For completeness and reproducibility purposes, the R code is accessible at https://github.com/mcampi111/c-VEMPs-in-healthy-children-bone-vs-air-conduction-normative-values. Normative values according to age were estimated using Stata/IC 10.0 for Windows (StataCorp LLC, College Station, TX, United States). Significance level was set at 5%; all tests were two-tailed.

## Results

AC c-VEMPs were obtained in all children and adult participants. Using the BC protocol, c-VEMPs were obtained in all but one of the children. This child, aged 15 years, had no BC responses but presented AC responses in both ears, as did 6 adult participants (aged 21, 43, 50, 56, 60, and 61 years). As a result, the success rate of the BC protocol was of 85.4% in adults. In both children and adults, there was no significant difference related to the ear side for all c-VEMP parameters: amplitude ratio, threshold, latencies (paired *t*-test, *p* > 0.05 for each characteristic). To test the equivalence of right and left ear responses in the same data set we performed Intra-class correlation (ICC) for AC and BC for all parameters (amplitude ratio, thresholds and latencies) to check for difference between ears. ICC is a descriptive statistic measuring units organized into groups, in our case, left vs. right ear for AC and BC, respectively. The ICC range for AC in the whole population was 0.84 [0.8;0.85] and for children 0.84 [0.78;0.89]. For BC the ICC range in the whole population was 0.86 [0.8;0.9] and for children 0.88 [0.84;0.91]. Hence, measurements from right and left ears were pooled in the same dataset for further analysis (Table 2).

**Table 2:** c-VEMP characteristics (amplitude ratio, P- and N-wave latency, threshold) as measured in children and adults.

	Age (years)	Ears	Amplitude ratio PN/EMG		P Latency (ms)		N Latency (ms)		Thresholds (dB nHL)	
			AC	BC	AC	BC	AC	BC	AC	BC
	Range	N	Mean (SD)	Mean (SD)	Mean (SD)	Mean (SD)	Mean (SD)	Mean (SD)	Mean (SD)	Mean (SD)
			Median (min;max)	Median (min;max)	Median (min;max)	Median (min;max)	Median (min;max)	Median (min;max)	Median (min;max)	Median (min;max)
CHILDREN	<1	16	0.8 (0.4)	1.7 (0.8)	12.9 (0.8)	13.0 (0.5)	18.1 (1.3)	19.7 (1.6)	78.0 (0.0)	74.8 (2.5)
			0.6 (0.4;1.6)	1.9 (0.5;2.8)	12.7 (11.2;14.2)	13.1 (11.8;13.8)	18.0 (16.0;20.8)	20.1 (17.0;22.2)	78.0 (78.0;78.0)	76.0 (71.0;76.0)
	1 to < 2	12	1.1 (0.8)	1.7 (1.0)	12.5 (0.5)	12.7 (0.6)	17.9 (1.0)	19.0 (1.2)	78.0 (0.0)	73.5 (3.5)
			1.1 (0.2;2.3)	1.9 (0.3;3.5)	12.5 (11.4;13.4)	12.6 (11.8;13.8)	18.0 (16.2;19.8)	18.7 (17.6;21.0)	78.0 (78.0;78.0)	73.5 (71.0;76.0)
	2 to < 3	10	1.7 (0.7)	2.6 (0.7)	12.7 (0.5)	13.0 (0.6)	18.6 (1.3)	20.2 (0.9)	78.0 (0.0)	73.5 (2.9)
			1.8 (0.4;3.0)	2.6 (0.8;3.3)	12.7 (12.0;13.6)	12.7 (12.4;13.8)	18.3 (17.0;21.6)	20.1 (19.0;21.4)	78.0 (78.0;78.0)	73.5 (71.0;76.0)
	3 to < 4	12	2.5 (0.9)	3.2 (0.8)	12.9 (0.5)	13.1 (0.6)	18.3 (0.7)	19.5 (1.7)	78.0 (5.2)	71.4 (6.9)
			2.8 (0.8;3.3)	3.2 (1.8;4.3)	12.9 (12.2;13.6)	12.9 (12.0;14.0)	18.2 (17.4;19.6)	20.0 (17.0;21.4)	78.0 (73.0;88.0)	71.0 (61.0;81.0)
	4 to < 5	10	2.0 (1.3)	1.8 (1.2)	13.0 (0.3)	12.9 (0.3)	19.2 (1.5)	19.2 (1.6)	71.8 (5.8)	72.3 (4.4)
			2.0 (0.4;4.3)	1.9 (0.4;3.8)	12.8 (12.6;13.4)	12.7 (12.6;13.4)	18.9 (17.6;22.4)	19.2 (15.4;21.2)	73.0 (63.0;78.0)	73.5 (66.0;76.0)
	5 to < 6	8	1.9 (0.4)	1.9 (0.3)	12.6 (0.6)	13.3 (0.9)	18.5 (0.8)	20.2 (2.2)	73.0 (0.0)	71.0 (0.0)
			2.0 (1.4;2.4)	2.0 (1.5;2.3)	12.5 (11.6;13.4)	13.3 (12.0;14.8)	18.8 (17.2;19.4)	19.2 (18.0;24.0)	73.0 (73.0;73.0)	71.0 (71.0;71.0)
	6 to < 7	8	1.3 (0.4)	1.4 (0.4)	12.6 (0.5)	12.7 (0.4)	18.9 (1.4)	18.7 (1.1)	79.3 (4.8)	76.0 (5.8)
			1.4 (0.7;1.9)	1.4 (0.8;2.1)	12.6 (12.0;13.2)	12.7 (12.0;13.2)	19.2 (17.0;20.8)	18.6 (16.8;20.2)	80.5 (73.0;83.0)	76.0 (71.0;81.0)
	7 to < 8	14	1.9 (0.7)	1.8 (0.7)	13.3 (0.9)	12.7 (0.7)	20.0 (1.5)	19.6 (0.9)	74.9 (7.5)	76.6 (10.5)
			1.8 (0.6;3.0)	1.9 (0.7;3.1)	13.0 (12.2;15.8)	12.8 (10.8;13.4)	19.6 (18.6;23.0)	19.5 (18.6;21.8)	73.0 (68.0;88.0)	71.0 (71.0;96.0)
	8 to < 9	22	2.0 (0.6)	2.0 (0.8)	13.2 (0.8)	12.9 (0.7)	19.7 (1.8)	19.5 (1.7)	75.2 (4.3)	73.5 (3.5)
			2.0 (0.8;3.7)	2.0 (0.6;3.2)	13.3 (11.6;14.4)	12.7 (11.8;14.0)	19.2 (17.0;23.2)	19.0 (17.2;23.8)	73.0 (68.0;83.0)	76.0 (66.0;76.0)
	9 to < 10	14	1.9 (1.1)	1.9 (0.7)	13.5 (0.9)	13.3 (1.0)	19.7 (1.0)	19.2 (1.0)	76.8 (2.3)	76.6 (1.8)
			1.5 (0.7;4.4)	1.8 (0.9;3.4)	13.4 (11.6;14.8)	13.1 (12.2;15.6)	19.5 (18.6;21.8)	18.8 (17.6;21.0)	78.0 (73.0;78.0)	76.0 (76.0;81.0)
	10 to < 11	20	1.8 (0.8)	1.6 (0.7)	13.5 (0.6)	13.1 (0.7)	20.0 (1.6)	19.3 (1.7)	75.0 (4.7)	76.8 (5.2)
			1.8 (0.6;3.6)	1.5 (0.5;3.1)	13.4 (12.6;14.8)	13.1 (12.0;14.4)	20.0 (17.6;23.4)	19.0 (16.8;23.4)	73.0 (68.0;88.0)	76.0 (66.0;86.0)
	11 to < 12	20	1.5 (0.9)	1.7 (1.1)	13.1 (0.8)	12.9 (0.8)	19.5 (1.4)	18.9 (1.3)	77.4 (6.0)	79.4 (5.1)
			1.2 (0.3;3.4)	1.4 (0.3;4.2)	13.1 (11.6;14.8)	12.7 (11.2;14.4)	19.8 (16.8;21.8)	19.0 (16.0;21.6)	78.0 (68.0;88.0)	76.0 (71.0;86.0)
	12 to < 13	22	2.1 (0.9)	2.0 (1.1)	13.5 (0.7)	13.3 (0.7)	20.0 (1.2)	19.5 (1.9)	76.8 (4.6)	78.0 (4.7)
			2.1 (0.6;4.0)	1.9 (0.3;4.0)	13.5 (11.6;14.8)	13.4 (11.0;14.6)	20.0 (16.4;22.4)	19.8 (14.6;23.6)	73.0 (73.0;83.0)	78.5 (71.0;86.0)
	13 to < 14	16	1.9 (0.8)	1.4 (1.1)	13.7 (0.7)	13.2 (0.7)	21.1 (1.6)	19.8 (1.4)	76.3 (3.9)	78.5 (4.0)
			1.9 (0.4;3.5)	1.1 (0.4;3.6)	13.9 (12.0;14.8)	13.2 (12.0;14.2)	21.0 (18.8;24.0)	19.9 (18.0;22.8)	75.5 (73.0;83.0)	76.0 (76.0;86.0)
	14 to < 15	22	1.6 (0.6)	1.6 (0.6)	13.7 (0.7)	13.6 (0.9)	20.4 (1.7)	19.8 (1.4)	78.0 (5.5)	81.3 (7.2)
			1.6 (0.7;2.6)	1.7 (0.4;2.6)	13.7 (12.6;14.8)	13.3 (11.6;15.6)	20.0 (17.6;24.8)	19.5 (18.0;24.0)	78.0 (68.0;88.0)	81.0 (71.0;96.0)
	15 to < 16	10	1.2 (0.3)	0.8 (0.4)	13.8 (0.8)	13.2 (0.5)	20.3 (1.9)	19.3 (0.8)	79.3 (4.4)	83.5 (6.0)
			1.2 (0.7;1.6)	0.7 (0.1;1.4)	13.6 (12.8;15.4)	13.3 (12.6;13.8)	20.2 (18.4;24.6)	19.3 (18.0;20.4)	80.5 (73.0;83.0)	83.5 (76.0;91.0)
ADULT	16 y.o to < 21 y.o	16	1.8 (1.0)	1.3 (0.7)	13.6 (0.4)	13.4 (0.7)	20.2 (1.5)	19.5 (1.2)	79.1 (4.5)	83.5 (5.1)
			1.5 (0.9;4.2)	1.1 (0.5;2.4)	13.6 (13.0;14.6)	13.4 (12.0;14.6)	19.8 (18.0;23.8)	19.6 (17.4;21.8)	78.0 (68.0;83.0)	81.0 (76.0;91.0)
	21 y.o to < 41 y.o	36	1.5 (0.8)	1.3 (0.5)	13.8 (1.0)	13.6 (0.8)	20.7 (2.0)	20.4 (1.6)	80.2 (6.7)	86.2 (7.6)
			1.4 (0.5;3.6)	1.2 (0.5;2.3)	13.8 (12.0;17.2)	13.5 (12.0;15.2)	20.3 (18.0;27.2)	20.5 (18.4;25.2)	78.0 (73.0;98.0)	91.0 (71.0;96.0)
	41 y.o to < 62 y.o	30	1.2 (0.6)	0.9 (0.5)	14.3 (0.8)	13.9 (1.1)	21.5 (1.6)	20.1 (1.7)	84.2 (3.8)	87.9 (5.2)
			1.0 (0.5;2.9)	1.0 (0.1;1.7)	14.4 (13.0;16.2)	13.9 (12.2;17.0)	21.4 (18.6;24.8)	20.3 (15.6;23.2)	83.0 (73.0;88.0)	86.0 (81.0;96.0)

In the infant group (0–1 year), one participant was 2 months old and eight were 6 to 12-months old.

To test the effect of testing position (Figures 3B,C) on EMG amplitude we compared the EMG level of children younger than 6 years and children older than 6 years (Welch’s two-sample t test). We found a significant difference in EMG amplitude between the two groups (younger than 6 years old and older than 6 years old) for AC (difference on average = 103.5 mV, *p* = 0.002) and a non significant difference in EMG amplitude between the two groups for BC (difference on average = 108.7 mV, *p* = 0.1349). However, the differences were partly corrected using EMG quantification from individual traces.

Normative data for each c-VEMP characteristic is presented next.

### Cervical vestibular evoked myogenic potential amplitude ratio (PN/EMG) for AC and BC stimulation

**Figure 4:**
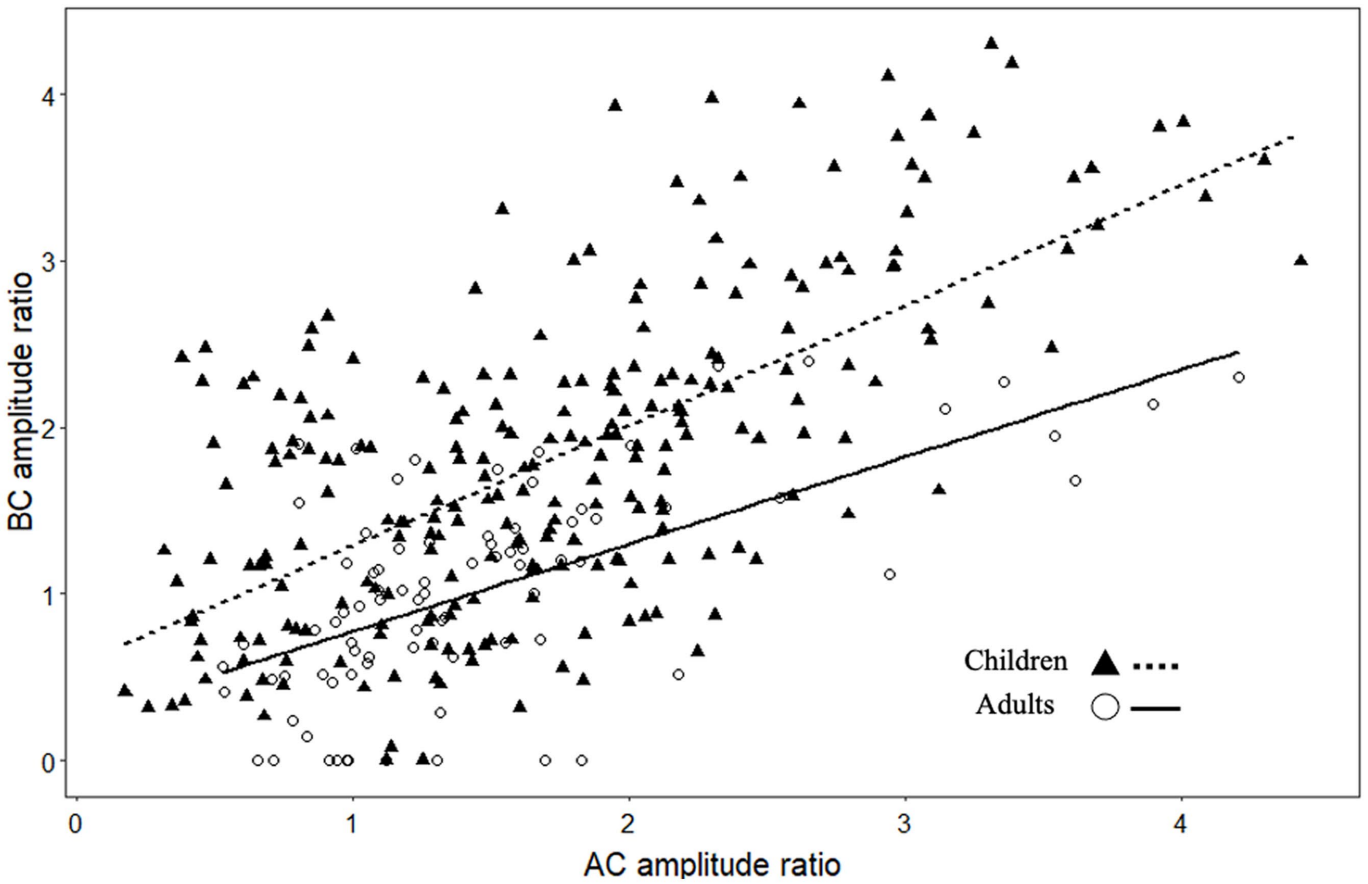
Scatterplot showing the relationship between c-VEMP amplitude ratios (P-N peak to peak amplitude/EMG amplitude) measured with air conduction (AC) and bone conduction (BC) stimulation.

**Figure 5:**
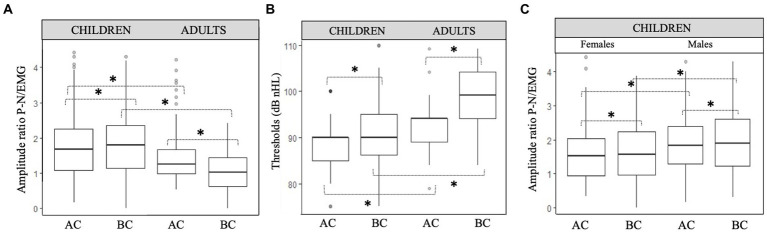
Comparison of c-VEMP characteristics in children and adults. **(A)** Amplitude ratio and **(B)** Thresholds (dB nHL) between children and adults and **(C)** amplitude ratio between males and females in children. Boxplots show median, lower and upper quartiles, and extreme values. Circles represent outliers. Paired *t* test with significant difference are indicated with a bracket and a star (*).

C-VEMP amplitude is known to present intra-individual and inter-individual variability (25). The amplitude ratios obtained for AC and BC were significantly correlated for children (*r* = 0.61, *p* < 0.001) and for adults (*r* = 0.59, *p* < 0.001; Figure 4). In the children group, BC amplitude ratio was slightly but significantly higher (*p* = 0.05) than AC amplitude ratio (for BC, median [IQR]: 1.8 [1.1–2.3] and for AC 1.6 [1.1–2.2]; Figure 5A). In contrast, for the adult group, the BC amplitude ratio (1.0 [0.6–1.4]) was significantly lower than for AC (1.3 [1.0–1.7]; *p* < 0.001; Figure 5A). Compared with the adult group, amplitude ratios in the children group were significantly higher for both AC (*p* = 0.01) and BC (*p* < 0.0001; Figure 5A).

**Figure 6:**
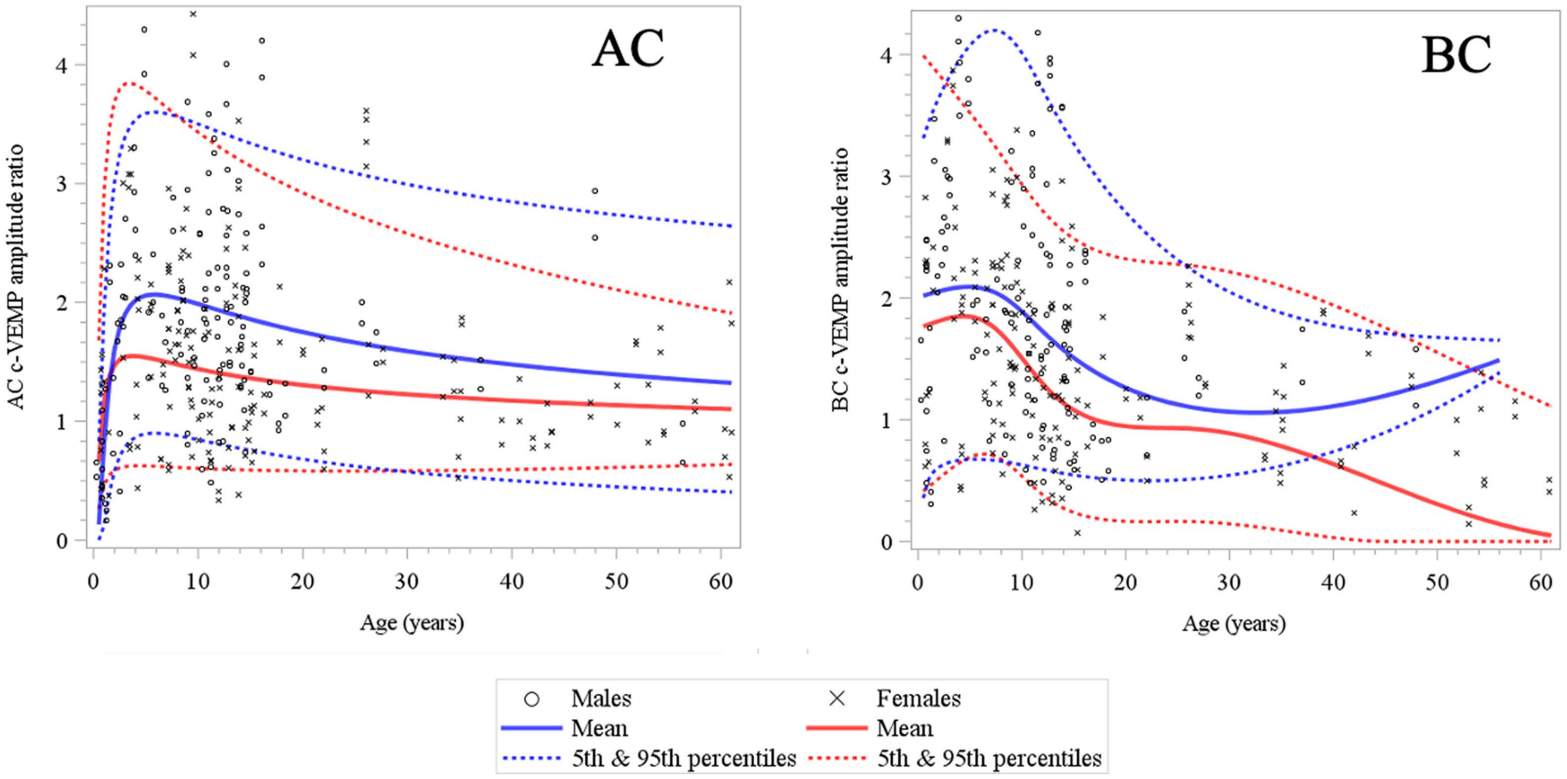
Changes in c-VEMP amplitude ratio for AC (air conduction) and BC (bone conduction) stimulation mode as a function of age and gender (6 months to 61 years) (26). Note that rapid changes occur mostly during early childhood (4–5 years) for AC and BC and there is after a slow and gradual decrease in all other age intervals. The tendency for an increase in older males of the mean curve in BC may be explained by the size and the asymmetric sex ratio distribution (11 males and 30 females) of the adult population.

**Figure 7:**
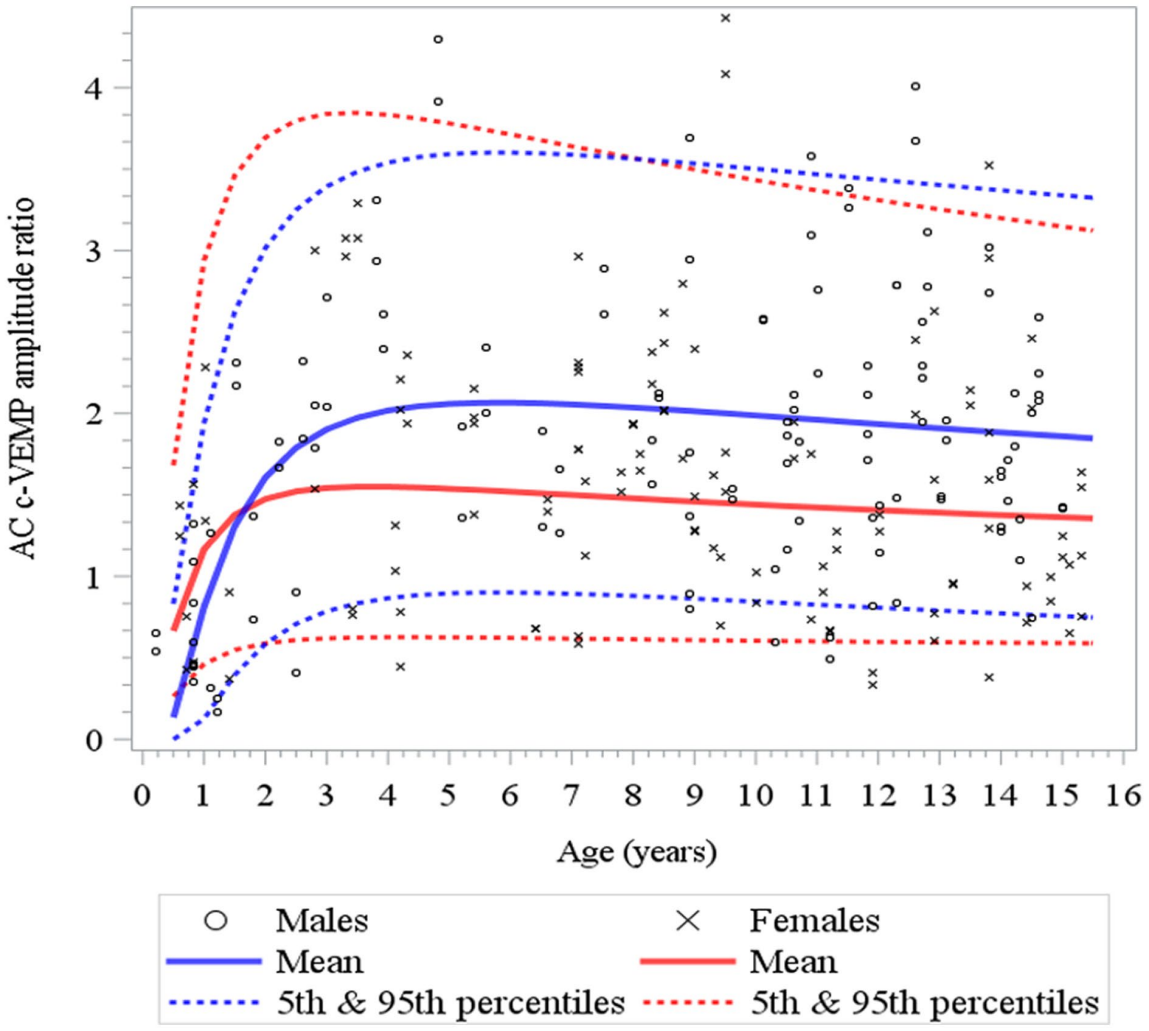
Changes in c-VEMP amplitude ratio for AC (air conduction) as a function of age and gender for children (6 months to 15 years of age) (26). After an initial rapid increase that lasts 4 to 5 years, but earlier in females (3 to 4 years) than in males (6 years) as shown also in Table 3. Note that although the mean of the amplitude ratio is higher for males than females, but this difference falls within the 95% confidence limits.

**Figure 8:**
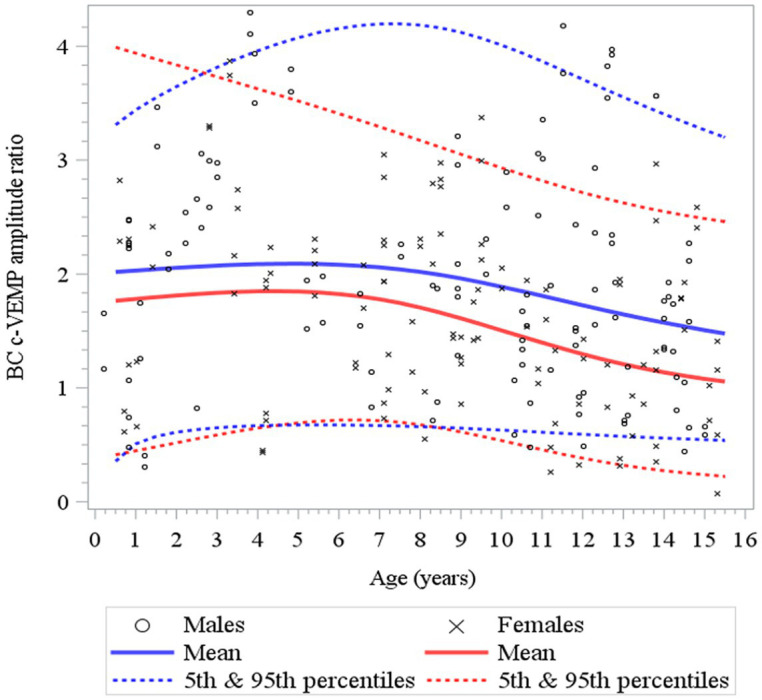
Changes in c-VEMP amplitude ratio for BC (bone conduction) stimulation as a function of age and gender for children (6 months to 15 years of age) (26). For BC the model shows that data are relatively stable function of age with a small increase during the first 4 to 5 years followed by a slow decline. Note also that the mean amplitude ratio is higher in males than in females.

In children, the amplitude ratio for males was significantly higher (median [IQR]:1.8 [1.3–2.3]) than in females (1.5 [0.9–2.0]; *p* = 0.02) for AC, as it was for BC (for males 1.9 [1.2–2.6] and for females 1.6 [1.0–2.2]; *p* = 0.03; Figures 5C, 6–8).

Here, to establish normative ranges for c-VEMP parameters by age and sex, we used data from the whole population (26) (Figure 6 for AC and BC). The Royston and Wright model relies on the age-specific reference interval method and estimates extreme quantile curves (5th, 10th, etc. percentiles). This provided the 5 and 95% confidence limits within which amplitude ratios can be considered normal for AC and BC for children (Figure 7; Table 3 for AC and Figure 8; Table 4 for BC). The measurements of the youngest patient (2, 5 months of age) were excluded as outliers. For AC, the mean and confidence limits for amplitude ratio increases rapidly for both sexes until the age of 4 years (for females) and 6 years (for males) and decrease progressively with age (Figure 7; Table 3). For BC, the changes are not as pronounced as for AC, and the mean and confidence limits increase slowly until 4 to 5 years of age for both sexes (slightly earlier for females than for males), then gradually decrease with age (Figure 8; Table 4).

**Table 3:** Normative values for air conduction (AC) c-VEMP amplitude-ratio as modeled for age-specific reference intervals using the Royston and Wright method (26).

	Males				
Age	5th percentile	10th percentile	Mean	90th percentile	95th percentile
1 year	0.13	0.23	0.81	1.66	1.94
2 years	0.59	0.78	1.61	2.67	3.02
3 years	0.79	1.00	1.90	3.03	3.39
4 years	0.87	1.09	2.02	3.17	3.54
5 years	0.90	1.12	2.06	3.22	3.59
6 years	0.90	1.12	2.07	3.23	3.60
7 years	0.89	1.12	2.06	3.22	3.59
8 years	0.88	1.10	2.04	3.20	3.56
9 years	0.86	1.08	2.01	3.17	3.53
10 years	0.85	1.06	1.99	3.14	3.50
11 years	0.83	1.04	1.96	3.11	3.47
12 years	0.81	1.02	1.93	3.07	3.44
13 years	0.79	1.00	1.91	3.04	3.40
14 years	0.77	0.98	1.88	3.01	3.37
15 years	0.76	0.96	1.86	2.98	3.34
	Females				
	5th percentile	10th percentile	Mean	90th percentile	95th percentile
1 year	0.46	0.57	1.17	2.40	2.94
2 years	0.59	0.72	1.47	3.02	3.69
3 years	0.62	0.76	1.54	3.14	3.84
4 years	0.63	0.77	1.55	3.14	3.83
5 years	0.63	0.76	1.54	3.10	3.78
6 years	0.62	0.76	1.52	3.05	3.71
7 years	0.62	0.75	1.50	2.99	3.64
8 years	0.61	0.74	1.48	2.94	3.57
9 years	0.61	0.74	1.46	2.88	3.50
10 years	0.60	0.73	1.44	2.83	3.43
11 years	0.60	0.73	1.42	2.79	3.37
12 years	0.60	0.72	1.41	2.74	3.31
13 years	0.60	0.72	1.39	2.70	3.25
14 years	0.59	0.71	1.38	2.66	3.20
15 years	0.59	0.71	1.36	2.62	3.15

Note that maximum amplitude ratio value of the mean and the 95 percentiles are, respectively, for male 2.07 and 3.60 at 6 years and for females 1.55 and 3.84 at 4 and 5 years.

**Table 4:** Normative values for bone conduction (BC) c-VEMP amplitude-ratio as modeled for age-specific reference intervals using the Royston and Wright method (26).

	Males				
Age	5th percentile	10th percentile	Mean	90th percentile	95th percentile
1 year	0.51	0.84	2.03	3.15	3.44
2 years	0.61	0.90	2.06	3.29	3.65
3 years	0.65	0.92	2.07	3.42	3.82
4 years	0.67	0.93	2.09	3.52	3.96
5 years	0.67	0.93	2.09	3.60	4.08
6 years	0.67	0.92	2.08	3.65	4.16
7 years	0.67	0.91	2.06	3.66	4.20
8 years	0.66	0.89	2.02	3.64	4.19
9 years	0.65	0.87	1.96	3.57	4.12
10 years	0.63	0.84	1.89	3.46	4.01
11 years	0.61	0.81	1.81	3.33	3.87
12 years	0.59	0.78	1.73	3.19	3.71
13 years	0.57	0.75	1.65	3.05	3.55
14 years	0.56	0.73	1.57	2.92	3.40
15 years	0.55	0.70	1.51	2.79	3.27
	Females				
	5th percentile	10th percentile	Mean	90th percentile	95th percentile
1 year	0.45	0.67	1.78	3.39	3.94
2 years	0.52	0.74	1.81	3.33	3.84
3 years	0.59	0.81	1.84	3.26	3.73
4 years	0.65	0.86	1.85	3.19	3.63
5 years	0.69	0.90	1.85	3.11	3.52
6 years	0.72	0.92	1.82	3.02	3.41
7 years	0.71	0.91	1.78	2.92	3.29
8 years	0.68	0.87	1.71	2.81	3.17
9 years	0.61	0.80	1.61	2.70	3.05
10 years	0.54	0.71	1.51	2.58	2.93
11 years	0.46	0.62	1.40	2.47	2.82
12 years	0.38	0.54	1.30	2.36	2.72
13 years	0.32	0.47	1.21	2.27	2.63
14 years	0.27	0.42	1.14	2.19	2.55
15 years	0.24	0.37	1.08	2.13	2.49

Note that maximum amplitude ratio value of the mean and the 95 percentiles are, respectively, for male 2.08 and 4.20 at 6 years of age and for females 1.85 and 3.94 as soon as 1 year.

**Figure 9:**
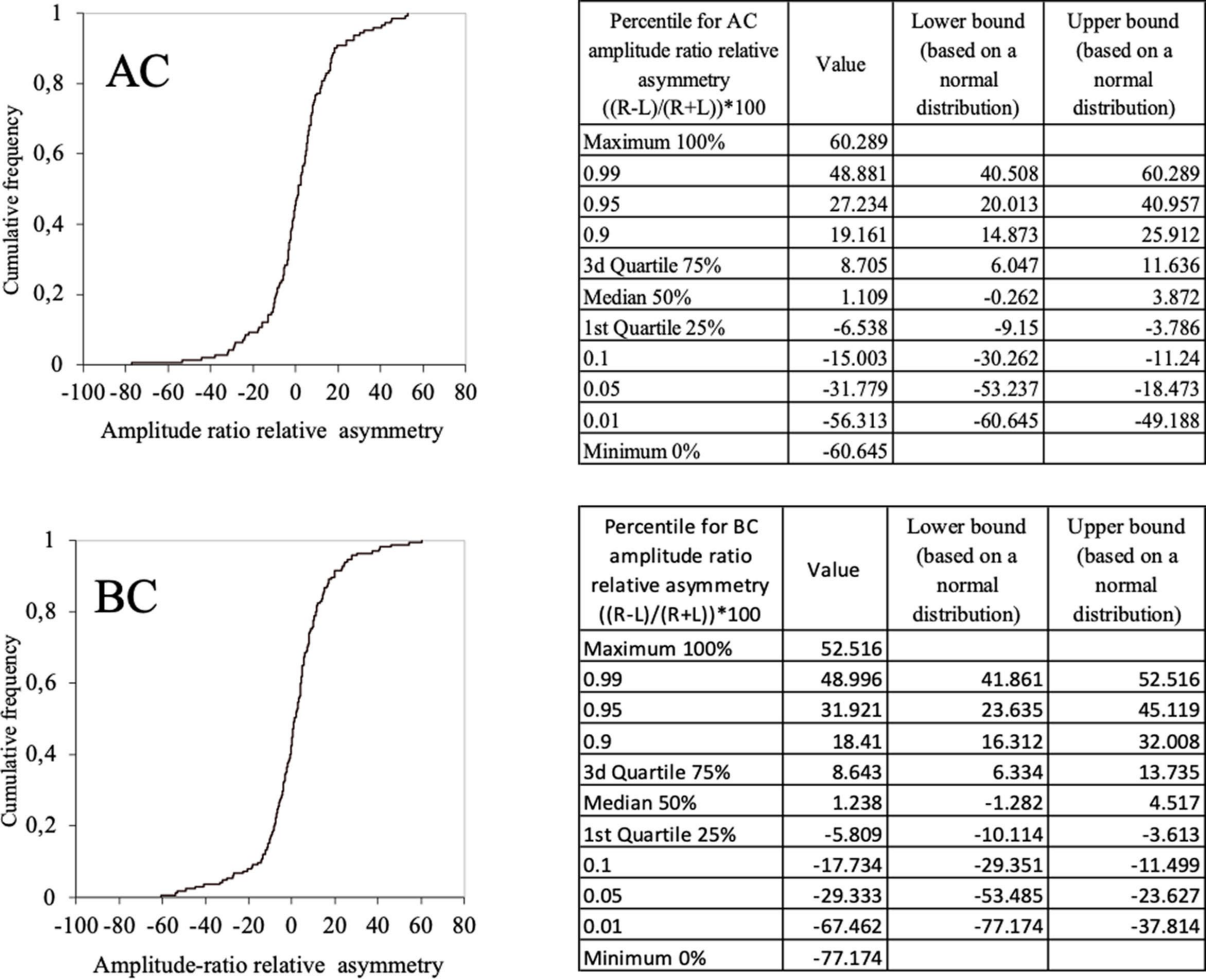
Normal values of c-VEMP amplitude ratio relative asymmetry for AC and BC for the whole population (children and adults) (cumulative histogram and tables). Relative asymmetry was calculated as follow: (((Right–Left)/(Right+Left))*100). For AC the 5–95% confidence limit values of the relative asymmetry range from [–32, 27%]. For BC the 5–95% confidence limit values of the relative asymmetry range from [–29, 32%].

In the whole population (children and adults) the relative asymmetry of the amplitude ratio in the 95% confidence limits ranged between [−31, 27%]. for AC (Figure 9-AC) and [−29, 32%] for BC (Figure 9-BC). Therefore, the magnitude for relative asymmetry of the amplitude ratio of 32% or less can be considered as limit for of normal values both AC and BC.

### c-VEMP thresholds for AC and BC stimulation

In children, c-VEMP threshold was negatively correlated with amplitude ratio for both AC (*r* = −0.52, *p* < 0.001) and BC (*r* = −0.46, *p* < 0.001). Thresholds between BC and AC (as well as for amplitude ratio) were significantly correlated in both children (*r* = 0.56, *p* < 0.0001) and adults (*r* = 0.50, *p* < 0.0001). Thresholds increased significantly with age (for AC: *r* = 0.37, *p* < 0.001; for BC: *r* = 0.64, *p* < 0.001; Table 2). Mean thresholds were significantly higher in adults than in children (*p* < 0.001) for both AC (children: 88 ± 5 dB nHL; adults: 93 ± 6 dB nHL; mean ± SD) and BC (children: 86 ± 6 dB nHL; adults: 96 ± 7 dB nHL; Figure 5B; Table 2). These differences are minor, since they are on the order of the 5 dB step used for threshold determination here. AC thresholds were significantly lower in males than females (mean ± SD: 85 ± 5 dB nHL for males, and 87 ± 5 dB nHL for females; *p* = 0.015) but, again, this difference is negligible. There was no significant difference for BC thresholds between males and females (mean ± SD: 86 ± 5 dB nHL for both). In children, the interaural asymmetries of the c-VEMP thresholds were inferior or equal to 5 dB.

### Cervical evoked myogenic potential P- and N-wave latencies for AC and BC stimulation

**Figure 10:**
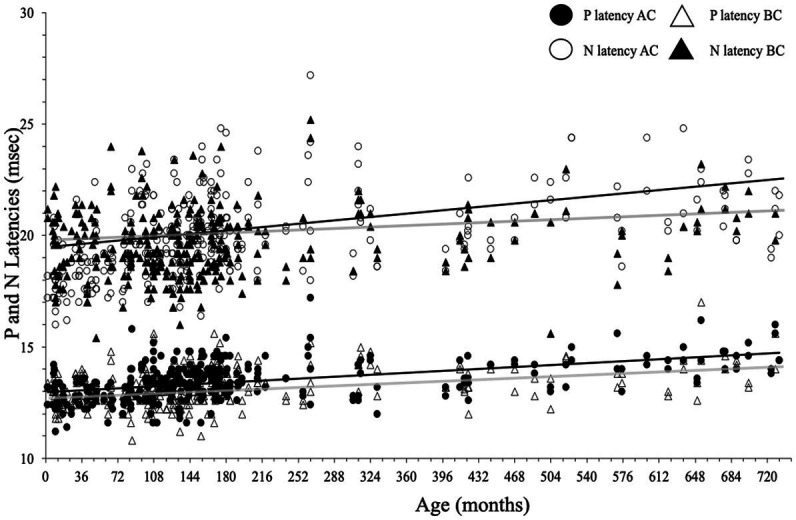
P- and N-wave latencies as a function of age for air conduction (AC) and bone conduction (BC). Regression lines (Spearman) are gray for BC and black for AC.

P and N latencies were positively correlated with age for both AC and BC (P latency for AC: *r* = 0.24, *p* < 0.001, and for BC: *r* = 0.13, *p* < 0.001; N latency for AC: *r* = 0.26, *p* < 0.001 and for BC: *r* = 0.03, *p* < 0.001; Figure 10; Table 2). The difference between the mean value of P and N latency between the youngest age group (<1 year) and the oldest age group (41–62 years) was larger for AC (1.4 msec for P latency and 3.4 msec for N latency) than for BC (0.9 msec for P latency and 0.4 s for N latency; Table 2).

#### P-wave latencies

In children, P-wave latency was significantly different (*p* = 0.01) between BC and AC (median [IQR] = 13.0 [12.6–13.6] msec for BC and 13.2 [12.6–13.8] msec for AC) as it was in adults (for BC: 13.5 [13.0–14.2] msec and for AC 14.0 [13.4–14.4] msec), but this difference was not clinically relevant. The P latency is shorter in children than in adults for both AC and BC (both *p* < 0.001; Figure 10) and the difference between youngest and oldest groups was 1.4 msec for AC and 0.9 msec for BC. No effect of sex was found for c-VEMP P latency either in children or adults (both *p* > 0.05).

#### N-wave latencies

There was no significant difference (*p* = 0.69) in N-wave latency between BC and AC (median [IQR] = 19.3 [18.4–20.4] msec for BC and 19.4 [18.4–20.5] msec for AC). In adults, the difference in N latency between BC and AC was significant (*p* = 0.001), but this is not clinically relevant (median N latency [IQR] for BC: 20.2 [19.0–21.0] msec and 20.5 [19.6–22.0] msec for AC; Figure 10). In children, N latency was significantly shorter than in adults both for AC and BC (*p* < 0.001) and the difference between youngest and oldest groups was 3.4 msec for AC and 0.4 msec for BC. No effect of sex was found for c-VEMP N latency either in children or adults (*p* > 0.05).

## Discussion

The present study validates AC and BC as stimulation to obtain equivalent c-VEMP responses in children. We provide normative data as a function of age and sex applicable in clinics for diagnosis. Significant differences observed between males and females and children and adults will be discussed.

Cervical vestibular evoked myogenic potentials characteristics differed between children and adults, independently of the stimulation mode (BC or AC): children had higher c-VEMP amplitude ratios, lower thresholds, as well as shorter P- and N-wave latencies compared to adults. Modeling showed that c-VEMP amplitude ratios changed with age during childhood, more for AC than for BC. AC amplitude ratio increases rapidly during the 4–6 years (with a maximum reached earlier in females) then gradually decreases from the age of 7 years through childhood and adulthood. BC amplitude ratio increases slightly during the first 4–6 years for both sex and then slowly decrease through childhood and adulthood. Here, the range of age for the normative values covers gaps between previous studies that included either infant younger than 2 years of age with only AC (17, 18), or with only BC only (20, 19), or older children belonging to scattered age groups (12, 13, 22, 23). In addition, the present study with a larger population provides more reliable values and better suited methods to the pediatric population.

Consistent with a recent study (12), BC and AC c-VEMPs amplitude ratios in children were higher than in adults. Another study in subjects older than 5 years (10 aged 5–10 years, 11 aged 11–18 years, and 11 aged 23–39 years) reported a significant decrease of c-VEMP amplitude with age for BC, but no correlation with age for AC (22). This is also compatible with the present study when considering the same age ranges.

As expected, c-VEMP amplitude ratio was negatively correlated with threshold for AC and BC, and mean threshold values were lower in children than in adults. This is consistent with Rodriguez report where children aged 4–9 years had lower mean AC c-VEMP thresholds than adolescents and young adults (13). We used modeling to describe the changes in the AC and BC c-VEMP amplitude ratio as a function of age. Compared to AC, changes in the amplitude ratio for BC in childhood are smaller (after high values maintained during 4–7 years), and the decrease is also progressive through adulthood. The decrease in c-VEMP amplitude after the age of 7 years could be related in part to the change in test position applied around the age of 6 years, because EMG amplitude may differ across positions. Although contraction differences were corrected using EMG quantification from individual traces, we cannot rule out a small effect of position on changes in amplitude ratio with age.

Multiple factors can impact the c-VEMP during childhood like bone structural changes and maturation of the vestibulospinal pathway.

Previous research from our group found that vestibulo-ocular response amplitude also increases rapidly during the first years of life, reaching a maximum around the age of 6 years before decreasing slowly (29). Here, AC and BC c-VEMP increase similarly during the first 4–6 years. This could suggest that vestibulo-spinal and vestibulo-ocular pathways may follow parallel maturational processes.

Cranial anatomical changes occurring during growth in childhood may also explain some changes of the c-VEMP responses with age.

The smaller size of the external meatus in very young children compared to adults (30) could impact the intensity of the stimulation delivered or by AC or by BC. With growth during childhood, the external auditory canal increases in size, which alters its resonance frequency; as a result, for a given AC intensity level stimulation, children receive a sound pressure level in the auditory canal that is 3 dB higher than adults (13). For Bone conduction the small size of the external canal in young children may induce a small occlusion effect that is maximal at low-frequency stimulation and increase the BC stimulation sound pressure level (31). BC stimulation is also known to recruit a greater number of otolith hair cells (from the saccule and utricle) than AC stimulation, which primarily stimulates hair cells from the saccule (8). This could explain why the BC amplitude ratio is higher than the AC amplitude ratio. The maximum amplitude ratios values for AC within the confidence limits are reached earlier in females than males (♂: 6 years; ♀: 4 years). This could be related to the results of Eby and Nadol’s investigation on temporal bone growth using CT scans (32). They reported that bone growth proceeds in two phases (rapid growth followed by a more gradual growth that continuous after childhood), and that the maximum bone thickness and mastoid pneumatization is reached for females earlier than in males. It is of note that, in children, c-VEMP amplitude ratios for both AC and BC were higher in males than in females irrespective of age. Patterson et al. (12) reported for BC a sex effect, but with lower amplitude ratio for males; however, his finding could have been biased by the sex ratio of 0.75 of their population (rather that 1.03 here in children) and a different age distribution between males and females (12).

In the present study, the success rate of both AC and BC c-VEMP recording in children reached 100%. In contrast, the success rate of BC c-VEMP recording dropped in adults and after 15 years of age to 85.4%. Similarly, higher mean threshold values for BC compared to AC were found in children and adults, however only in adults was the difference greater than the recording protocol sensitivity (5 dB-step). Several authors reported in adults a lower response rate for BC than AC as well as a decrease of the response rate with age more pronounced for BC than AC (6, 16, 21). Interestingly, we observed in 3 of the adult subjects who were tested longitudinally at several years interval, a progressive bilateral decrease of the BC amplitude ratio and finally a loss of response to BC at 96 dB nHL while their response to AC remained normal and the subjects remained healthy with no sign of otolith dysfunction (see Supplementary material). Whether or not BC c-VEMP can be obtained in individual adult subjects this may be related to inter-subject variability in mastoid anatomical characteristics. Changes in cranial bone mechanical properties according to age are likely to explain why BC c-VEMP amplitude ratio decrease and thresholds increase with age, as well as why there is an increasing frequency of normal subjects (from the age of 15 years) with an absence of c-VEMP response to BC but normal c-VEMP responses for AC. An increase in bone cortical thickness could decrease the efficiency of BC conduction as would higher mastoid pneumatization (32, 33).

P- and N-wave latency increase with age, but differences were small over the wide age range herein. These results are coherent with previous studies. For example, Kelsch reported that AC c-VEMP latencies are shorter in children aged 3–5 years compared to older ones (up to 11 years) (23). However, studies conducted in newborns found longer P- and N-wave latency in pre-term than full-term neonates (18), and longer P- and N-wave latency in full-term neonates than adults (17). Taken together, these findings suggest that the maturation of the vestibulospinal pathway mostly occurs during the first months of life and may even be triggered before birth.

Different protocols have been proposed to record c-VEMPs in children (12, 13, 17–20, 22). However, their results are difficult to compare because some were obtained with BC stimulation and others with AC stimulation. Among the studies cited above only Martens et al. (19) used, as here, the normalization method based on EMG quantification from individual trace EMGs. This was recently reported as the best approach by van Tilburg et al. (25) who compared all currently available normalization techniques. This normalization method overcomes the dependency of c-VEMP amplitude on neck muscle amplitude contraction, and thus decreases the high intra- and inter-individual variability of the EMG (14).

Consistent with several recent publications, BC stimulation here delivered 750 Hz short tone bursts with a B71 RadioEar vibrator applied on the mastoid. However, in case of the absence of AC c-VEMP responses, Patterson et al. (12) recommend the use of a B81 vibrator for BC stimulation. Although this vibrator provides 5 dB more maximal output than the B71 (34), there was no significant difference in c-VEMP amplitude induced by B71 versus B81 (35). Thus, using it would be unlikely to yield different results than those reported here. Another issue concerns the frequency of the stimulus (750 Hz) used. The comparison between AC and BC c-VEMP responses required equal stimulation frequencies (11). Reported that the largest c-VEMP amplitudes were found for a frequency range stimulation of 500–1,000 Hz. Almost all reported studies have used 500 Hz (7). But we used 750 Hz which may present several advantages. For instance, Piker et al. (21), found the largest c-VEMP peak-to-peak amplitudes at 750 Hz. There were also concerns about the negative effect of high intensity stimulation on the hearing function (36, 37) and it is of note that Rodriguez et al. (15) reported that 750 Hz-tone burst in case of narrow external auditory canals (such has found in children) provides a 2 dB SPL lower sound exposure level compared to 500 Hz. In addition, to reduce acoustic energy exposure, the use of short tone-burst stimuli was recommended (6, 38) with the smallest number of trials possible (13). Here, the number of stimulations necessary to obtain reproducible c-VEMP responses was only around 25 repetitions for children and 50 repetitions for adults.

In our pediatric population free of any conductive disorders, there were differences in c-VEMP threshold and latency between BC and AC, but these were negligible. This indicates that BC and AC can be used indifferently in subjects under 15 years of age. In contrast, significant and clinically relevant differences were seen in adults between BC and AC for all c-VEMP characteristics, especially BC amplitude ratio and thresholds.

The present study has a few limitations. Possibly the most important is that, although the population included was large enough to cover a wide range of age intervals, only one participant was under 6 months of age. Also, the normative BC data were obtained with a single type of bone transducer and a short 750 Hz-tone burst and no other bone transducers, e.g., impulse hammer or mini-shaker to compare.

Another question remains here as to why some subjects (from age 15 onwards) loose the BC response while retaining normal AC responses (as reported in 3 adult subjects of our study). Further longitudinal study combining c-VEMP recordings and radio-morphological data in healthy subjects will test our hypotheses. A more general aspect is to overcome the fact that each subject was tested at only one age while there is a large intra- and inter-subject variability in amplitude ratio. Therefore, a modeling approach was needed to estimate age- and sex-specific normative values and provide data for future studies and clinical use. In interpreting the changes in c-VEMP amplitude with age, we could not completely exclude the effect of testing position on EMG amplitude, although normalization using EMG quantification from individual traces probably reduced its impact. In conclusion, normative data from pediatric c-VEMP recordings here provide boundary criteria for normality for amplitude ratios, thresholds, and latencies. These criteria for children are as follows:

- Amplitude ratio fitting in the 95% confidence interval established with a model depending on sex and age and provided in tables,
- Relative interaural amplitude ratio asymmetry less than 35%
- Threshold inferior or equal to 95 dB HL (about 85 dB nHL) with an interaural threshold difference inferior or equal to 5 dB,
- P-wave latency ranging between 12.6–13.8 msec both for BC and AC.

## Conclusion

In children aged 6 months to 15 years, c-VEMPs can be obtained using an adapted pediatric protocol, either by BC (B71 vibrator) or by AC stimulation in response to short 750 Hz-tone bursts. After an initial increase during the first 4–6 years of age (much more pronounced for AC than BC), the c-VEMP amplitude ratio gradually decreases after the age of 7 years (for both AC and BC). Amplitude ratios depends on sex, with higher amplitude ratios in males than in females for both modes of stimulation (an effect more pronounced for AC than BC). These results suggest that BC c-VEMPs represent a valid alternative to AC c-VEMPs in the vestibular assessment battery in children up to 15 years of age and should prove essential in pathologies with impaired air conduction.

## Data availability statement

The raw data supporting the conclusions of this article will be made available by the authors, without undue reservation.

## Ethics statement

The studies involving human participants were reviewed and approved by (Comité de Protection des Personnes, Ile de France; # 96048). Written informed consent to participate in this study was provided by the participants’ legal guardian/next of kin.

## Author contributions

SW-V designed and performed the experiments, collected the data, and wrote the manuscript. SW-V, MC, PB, and HT-V performed the statistical analyses and their graphical presentation in the manuscript. SW-V and HT-V analyzed the data and wrote the manuscript. All authors contributed to the article and approved the submitted version.

## Conflict of interest

The authors declare that the research was conducted in the absence of any commercial or financial relationships that could be construed as a potential conflict of interest.

## Publisher’s note

All claims expressed in this article are solely those of the authors and do not necessarily represent those of their affiliated organizations, or those of the publisher, the editors and the reviewers. Any product that may be evaluated in this article, or claim that may be made by its manufacturer, is not guaranteed or endorsed by the publisher.
