# Supplementary material for: Cervical vestibular evoked myogenic potentials in healthy children: Normative values for bone and air conduction
Source: Front Neurol. 2023 Apr 18;14:1157975. doi: 10.3389/fneur.2023.1157975 (PMC10152971; doi:10.3389/fneur.2023.1157975)
Supplement: Supplementary file 1 [file Data_Sheet_1.docx]

Supplementary Material: Changes with age of the c-VEMP amplitude ratio (PN/EMG) in 3 adults from our study who were tested several years apart. The c-VEMP response to BC progressively decreases bilaterally with time (subject S2) and becomes undetectable at 96 dB nHL with BC on both sides (subjects S2 and S3) while AC responses remain normal and the subjects remain healthy with no evidence of ear dysfunction.
